# The histaminergic control of the iridal vascular tone in rats and its influencing by topical administration of olopatadine and ranitidine

**Published:** 2019

**Authors:** Dragoș-Constantin Luncă, Horia Păunescu, Ovidiu Mușat, Ion Fulga

**Affiliations:** * Department of Pharmacology and Pharmacotherapy, Faculty of Medicine, „Carol Davila” University of Medicine and Pharmacy, Bucharest, Romania; ** ”Dr. Carol Davila” Central Military Emergency University Hospital, Bucharest, Romania

**Keywords:** histamine, olopatadine, ranitidine, arterioles, venules, iris, rat

## Abstract

**Objective.** We evaluated the histamine’s role in regulating the iris vasomotricity in rats, using as a research tool topical olopatadine, a selective H1 blocker, which is indicated for the treatment of allergic conjunctivitis and ranitidine, a selective H2 blocker mainly used for the treatment of peptic ulcer disease.

**Methods.** Two groups of six Wistar rats anesthetized with ketamine 200 mg/kg body weight were used. They received distilled water in conjunctival instillations, initially and after 5 minutes, olopatadine 2.5 mmol/ l for the first group, respectively ranitidine 2.5 mmol/ l for the second group. The changes of the iris arteriolar and venular diameters were recorded.

**Results.** Both olopatadine and ranitidine produced statistically significant iridal arteriolar vasoconstriction and ranitidine determined statistically significant venuloconstriction, while distilled water did not produce any statistically significant effect.

**Conclusions.** There is a vasodilator histaminergic tone exerted through the histaminergic H1 and H2 receptors in the iris arterioles and, respectively, through the H2 receptors in the iridal venules. Olopatadine, a topical H1 antagonist used in the treatment of ocular allergies, may interfere with the humoral regulation of the iris arteriolar tone. Ranitidine, an H2 antagonist, decreased the diameter of the iris arterioles and venules, when administered topically in rats.

## Introduction

Histamine, an ubiquitous biogenic amine, that has multiple biological roles, is also found in the mammals’ eyes, where it possibly controls the ocular vascular tone [**1**]. The biological effects of histamine are produced by activating histamine receptors. Until now, according to IUPHAR (the International Union of Basic Pharmacology and Clinical Pharmacology), four histaminergic receptors have been described and have been labeled with arabic numerals, from H1 to H4, but the most studied until now are the H1 and the H2 receptors [**2**].

H1 receptor blockers, whose effects have been studied at the ocular surface, are levocabastine, olopatadine, alcaftadine, and others [**3**]. They are used in the symptomatic relieve of ocular allergies. Perennial allergic conjunctivitis and seasonal allergic conjunctivitis have low intensity symptoms and they could be treated non-pharmacological with artificial tears and pharmacological with OTC drugs (e.g. tetrahydrozoline). In moderate cases, antihistamines and/ or stabilizers of mast cell membranes are prescribed. In severe cases of allergic conjunctivitis such as atopic keratoconjunctivitis and vernal keratoconjunctivitis, it is often necessary to use glucocorticoids, non-steroidal anti-inflammatory, and/or immunomodulatory agents [**4**].

A Cochrane review of the treatment of perennial and seasonal allergic conjunctivitis with topical antihistamines and mast cell stabilizers, conducted by Castillo et al. in 2015, using several databases (data analysis up to 17 June 2014), identified 30 trials with over 4000 participants and 17 different drugs. Due to the variability in the quality and in the reporting of the studies, only a single meta-analysis could be made, which compared olopatadine with ketotifen, both topically administered. These were only short term treatments, from one to eight weeks. The bias risk was judged small and there were no serious problems with the safety of these medicines. The comparison of olopatadine-ketotifen was in favor of olopatadine [**5**].

Olopatadine is the most commonly used topical antihistamine in Romania and its therapeutical indication is the treatment of seasonal allergic conjunctivitis (Olopatadine’s Summary of Product Characteristics). Olopatadine belongs to the dual acting antihistamine drugs, being both a selective H1 receptor blocker and a stabilizer of mast cell membrane [**6**]. From a pharmacodynamic point of view, olopatadine has an affinity for H1 receptors approx. 1000 times higher than for H2 receptors and, respectively, approx. 4100 times higher than for H3 receptors. It has a greater selectivity for H1 receptors than other antihistamines such as pheniramine, antazoline, ketotifen and levocabastine [**7**]. Olopatadine lacks effect on alpha-adrenergic, dopamine and muscarinic type M1 and M2 receptors [**8**]. Administered topically in rabbits, olopatadine reached the highest concentrations in the cornea and bulbar conjunctiva and was approximately 10 times lower at the level of iris-ciliary body complex [**9**].

H2 receptor blockers are indicated for treating peptic ulcer disease in internal medicine. Ranitidine is one of the most used H2 antagonist in Romania, both in orally and intravenously route administration (Ranitidine’s Summary of Product Characteristics). In clinical practice, ranitidine administered together with a H1 receptor antagonists can also be used in treating some types of allergic reactions, such as anaphylactic shock or urticaria, because it blocks the effects of histamine, which has a major role in the pathogenesis of various types of allergic reactions [**10**]. In spite of these empirical use, systematic reviews did not identify randomized clinical trials demonstrating the efficacy of H2 antagonists in anaphylactic shock or urticaria [**11**,**12**].

## Methods

We used two groups of six Wistar male rats weighting between 300 and 350 grams, brought in the laboratory 4 days prior experiments. The rats had ad libitum access to food and water. The experiments took place in the daylight and we investigated only the right eye of the animals. The Ethics Committee of Bucharest’s “Carol Davila” University of Medicine and Pharmacy approved the experiments.

We used the following substances: 10% ketamine (CP-Ketamine 10%), distilled water, olopatadine, ophthalmic solution 1 mg/ ml (Opatanol 1mg/ ml, ocular drops, Alcon, UK), ranitidine, injectable solution 25 mg/ ml (Arnetin 50 mg/ 2 ml, injectable solution, Medochemie, Cyprus).

**Fig. 1:**
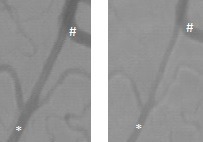
Grayscale images captured at 0 seconds, before instillation of distilled water (left image) and at 150 seconds after the instillation of 2.5 mmol/ l olopatadine solution (right image). *arteriole, # venule

Experimental procedure: We anesthetized the rats before the instillation of substance, using 10% ketamine (200 mg/kg body weight). After 15 minutes, the rats were in left lateral decubitus and the eyelids were opened manually to enhance the visualization of the eye. The right eye of each rat was recorded at 400X maximum magnification, using an optical system made of NIKON objective lens and a NAVITAR 1X Adapter, connected to an analog camera and an analog-to-digital video converter. A ring shaped cold light source was used for illumination. Magnification and lighting conditions were maintained constant during each experiment. Each eye was recorded for 11 minutes. For each iridal arteriole and venule analyzed, we made image captures and we measured the diameters of the arterioles and the venules at the specific moments of the 11 minutes recording: 0 seconds (t0), 120 seconds (t2), 180 seconds (t3), 210 seconds (t4), 300 seconds (t5), 420 seconds (t7), 480 seconds (t8), 510 seconds (t9), 600 seconds (t10), 630 seconds (t11). We administered distilled water at 30 seconds (t1) and at 330 seconds (t6), the 2.5 mmol/ l olopatadine solution was instilled for the first group, respectively the 2.5 mmol/ l ranitidine solution for the second one. Practically, the diameters of the iridal venules and arterioles were measured before the instillation of distilled water, at the beginning of the recording, then at 90 seconds, 150 seconds, 180 seconds, 270 seconds after the instillation of distilled water, and at 90 seconds, 150 seconds, 180 seconds, 270 seconds and 300 seconds after the instillation of 2.5 mmol/l olopatadine or ranitidine 2.5 mmol/l. The vessel’s diameters were measured in pixels at the intersection between a venule and an arteriole, the vessel which was located posteriorly at the intersection and which was greater in size was consider a venule and the vessel which was located anteriorly at the intersection and which was smaller in size was consider an arteriole. We used the software ImageJ 1.51j8 with the Diameter plug-in [**13**] to measure the arteriolar and the venular diameters on grayscale images (see **Fig. 1**). For every captured image at a specific moment and for each eye, we measured four arteriolar and four venular diameters. For every captured image specified above, we calculated the mean arteriolar / venular diameter and the relative diameter using the formula:

$$D_{rel}=\frac{D_{x}-D_{0}}{D_{0}}*100$$

(Formula 1)

where Drel was the relative change in diameter compared to the 0 second moment, Dx was the vascular diameter in pixels at a specific moment and D0 was the vascular diameter in pixels at 0 seconds. We calculated for each moment, the relative diameter, the standard error, and the statistical significance of the differences between this moment and the 0 seconds moment. We used the paired variant of t-student test (each eye was its own control). If p<0.05, the differences were considered statistically significant.

**Fig. 2:**
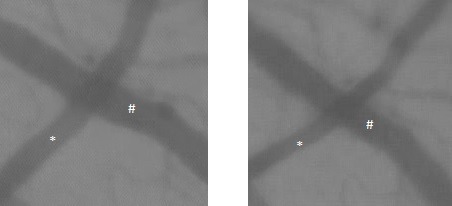
Grayscale images at 0 seconds, before instillation of distilled water (left image) and at 270 seconds after the instillation of 2.5 mmol/ l ranitidine solution (right image). *arteriole, # venule

## Results

**Fig. 3:**
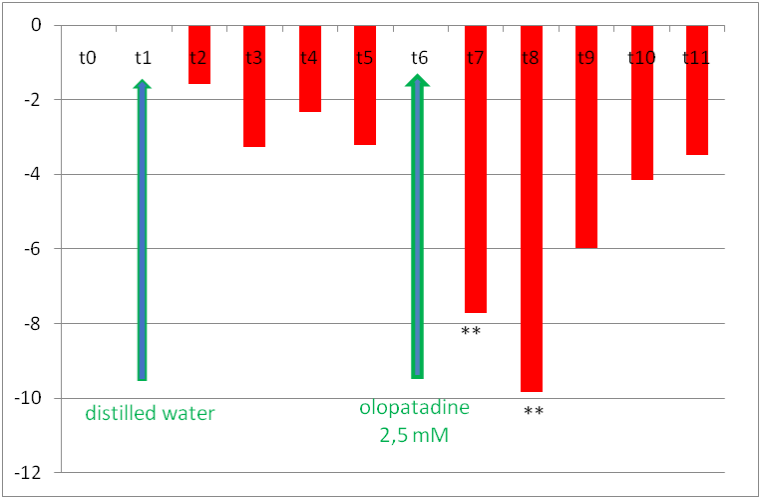
The changes of iridal relative arteriolar diameters in rat’s eye after administration of distilled water at 30 seconds (t1) and subsequently of 2.5 mmol/ l olopatadine, at 330 seconds (t6). The columns’ heights represent the relative vascular diameter values calculated with formula 1 (see materials and methods) (** p<0.05)

**Fig. 4:**
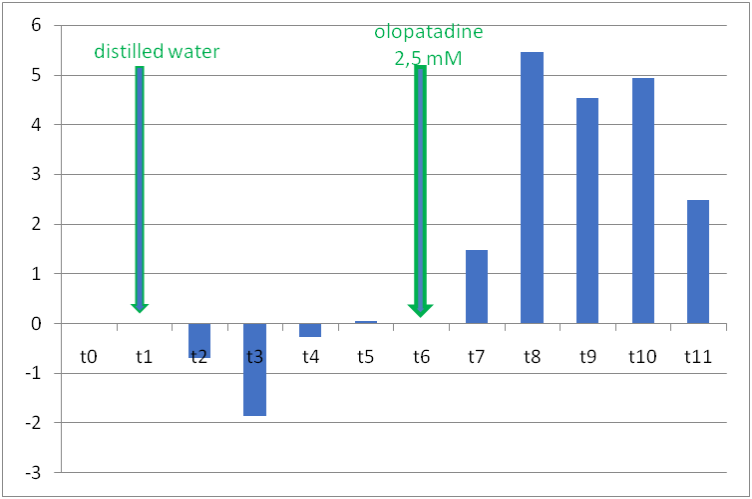
The changes of iridal relative venular diameters in rat’s eye after instillation of distilled water at moment t1 and subsequently of 2.5 mmol/ l olopatadine, at moment t6. The specific moments, at which the measurements were made, are presented on the horizontal. The columns’ heights represent the relative vascular diameter values calculated with formula 1 (see materials and methods) (** p<0.05)

For the first group (olopatadine), the evolution of the relative changes of vascular diameter is shown in **Fig. 3** for iridal arterioles and in **Fig. 4** for the iridal venules. Administration of 2.5 mmol/ l olopatadine after distilled water decreased the iridal arterioles diameters by 7.72% +/ - 2.9% at moment t7 and by 9.82% +/ - 2.82% at moment t8. These values were statistically significant in relation to moment t0, for p<0.05. 

2.5 mmol/ l olopatadine solution did not significantly change the relative diameter of iris venules. Distilled water did not significantly change the relative diameters of iris arterioles or venules.

**Fig. 5:**
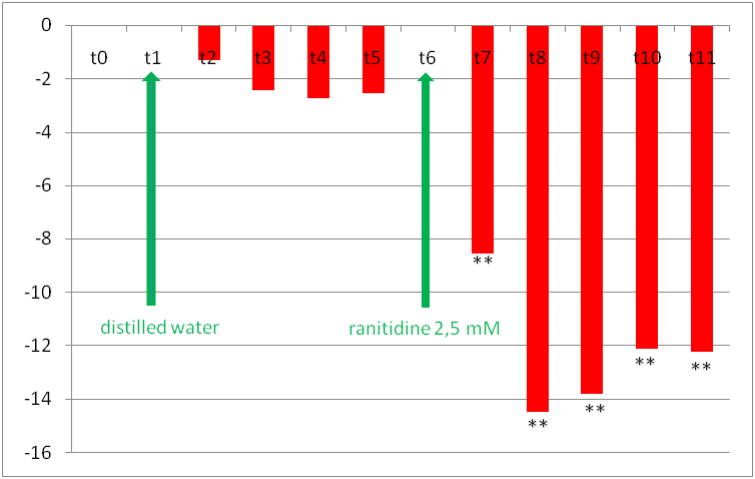
The changes of iridal relative arteriolar diameters in rat’s eye after instillation of distilled water at moment t1 and subsequently of 2.5 mmol/ l ranitidine, at moment t6. The specific moments, at which the measurements were made, are presented on the horizontal. The columns’ heights represent the relative vascular diameter values calculated with formula 1 (see materials and methods) (** p<0.05)

**Fig. 6:**
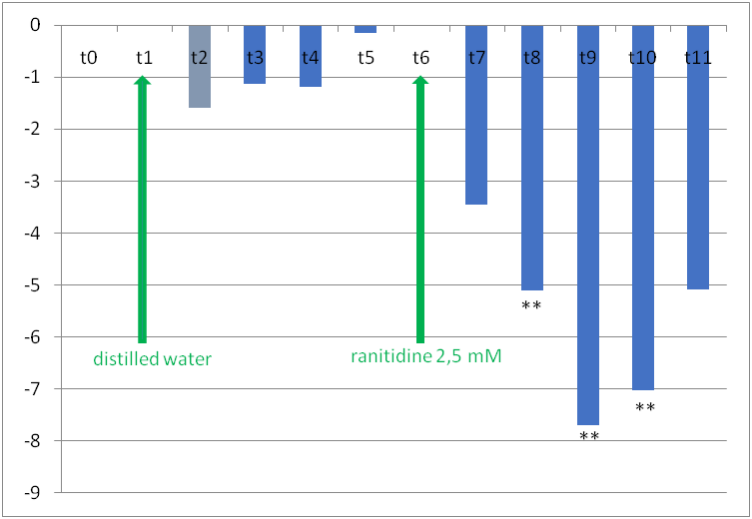
The changes of iridal relative venular diameters in rat’s eye after instillation of distilled water at moment t1 and subsequently of 2.5 mmol/ l ranitidine, at moment t6. The specific moments, at which the measurements were made, are presented on the horizontal. The columns’ heights represent the relative vascular diameter values calculated with formula 1 (see materials and methods) (** p<0.05)

For the second group (ranitidine), the evolution of the relative changes of vascular diameter is shown in **Fig. 5** for iridal arterioles and in **Fig. 6** for the iridal venules. On the iridal arterioles, ranitidine decreased their diameter by 8.53% +/ - 1.50% at moment t7, by 14.47% +/ - 2.36% at moment t8, by 13.77% +/ - 1, 31% at moment t9, by 12.11% +/ - 2.70% at moment t10 and by 12.22% +/ - 2.27% at moment t11. Distilled water did not significantly change the relative diameters of iris arterioles or venules.

On the iridal venules, ranitidine decreased their diameter by 5.09% +/ - 1.43% at moment t8, by 7.70% +/ - 1.69% at moment t9 and by 7.03% +/ - 2.58% at moment t10. Distilled water did not significantly change the relative diameters of iris arterioles or venules.

## Discussion

We wanted to verify if there is a histamine tonic regulation of iris vasomotricity, not a phasic one. The tonic control of a body structure involves the fulfillment of two conditions: the permanent existence of a minimum concentration of an endogenous substance at that level, namely an adequate density of receptors on which that substance acts as an agonist. If we get opposite effects to those of the agonists when administering only the antagonist, we can say that there is tonic control. If the antagonist does not produce an effect when given alone, but is able to prevent the effect of exogenous agonist, we can say that we have phasic control [**14**].

From the data presented above, it results that olopatadine produced a 7-10% decrease in iridal arteriolar diameter, compared with moment t0, an effect that lasted less than 5 minutes. Administration of olopatadine, which blocks H1-type receptors, has produced iridal arteriolar vasoconstriction. We can say that there is a histaminergic vasodilator tone produced via H1 receptors at the level of the iridal arterioles.

Olopatadine did not significantly modify the diameter of the iridal venules. This does not allow us to affirm, at least at this moment, that a histaminergic vascular tone is not achieved in iridal venules via type 1 histaminergic receptors.

Administration of ranitidine, which blocks H2 receptors, produced vasoconstriction in both the arterioles and the venules of the iris. From the data presented above, ranitidine produced a 12-14% decrease in the iridal arterioles compared with moment t0, an effect that lasted more than 5 minutes, and, in the iridal venules, produced a vasoconstriction of approximately 8-10%, an effect that lasted less than 5 minutes. These data suggest that there is a histaminergic vasodilatory tone exerted through H2 receptors in both iridal arterioles and venules. These results can be explained by the fact that ranitidine blocks a H2 - histaminergic vasodilatory tone, present in both iridal arterioles and venules.

Correlated with the results obtained in the olopatadine group and given that both olopatadine and ranitidine were used in equimolar concentrations, we can hypothesize that there is a difference between H1 and H2 receptor densities in the iridal venules, with a higher density of H2 receptors. The fact that we did not achieve statistically significant venular vasoconstriction when we administered olopatadine, does not allow us to make any assertion about the H1 receptor density in the venular territory. The fact that in the case of ranitidine, the vasoconstrictor effect was more intense and lasted longer than that of olopatadine, allows us to issue a second hypothesis, that the H2 receptor density was higher than the H1 receptor density in the iridal arterioles.

There is little data from literature regarding in vivo experiments using topical H1 or H2 antagonists. Very few have used ranitidine or other H2 blocker and practically there are no studies about the effect of olopatadine on the iridal vascular tone. Other H1 receptor blocking agents have been used, like diphenhydramine, pyrilamine and promethazine, but the results of these studies have as a disadvantage the lack of selectivity on histamine H1 receptors, whereas olopatadine is a selective H1 receptor antagonist [**7**,**15**]. Further studies are needed to elucidate the effects of histaminergic substances on iris vasculature, using combinations of exogenous histamine and histamine receptor blockers.

## Conclusions

1. A histamine vasodilator tone is present in both iridal arterioles and, respectively, iridal venules.

2. In the iridal arterioles, histamine exerts its vasodilatory tone, both via H1 receptors and H2 receptors.

3. In the iridal venules, histamine exerts its vasodilatory tone only via H2 receptors.

4. The stimulation of H1 and H2 receptor produces vasodilatation in the iridal arterioles, as it does in majority of vascular areas of the body.

5. The density of H2 receptors is likely to be greater than that of H1 receptors, both in the iridal arteries and in the iridal venules.

6. Ranitidine, a H2 antagonist, decreased the diameter of the iridal arterioles and venules, when administered topically in rats.

7. Olopatadine, a drug indicated for the treatment of eye allergies, may interfere with the humoral regulation of iridal arterial vascular tone.

**Compliance with ethical standards**

All procedures performed were in compliance with the ARVO Statement for the Use of Animals in Ophthalmic and Vision Research and with procedures approved by the Local Ethics Committee of “Carol Davila” University of Medicine and Pharmacy, Bucharest, Romania.

**Funding**

None.
